# Effects of Cool Water Supply on Laying Performance, Egg Quality, Rectal Temperature and Stress Hormones in Heat-Stressed Laying Hens in Open-Type Laying Houses

**DOI:** 10.3390/ani15111635

**Published:** 2025-06-02

**Authors:** Chan-Ho Kim, Woo-Do Lee, Se-Jin Lim, Ka-Young Yang, Jung-Hwan Jeon

**Affiliations:** Animal Welfare Division, National Institute of Animal Science, Rural Development Administration, Wanju 55365, Republic of Korea; woodo92@korea.kr (W.-D.L.); limsj818@korea.kr (S.-J.L.); y2k1983@korea.kr (K.-Y.Y.); jeon75@korea.kr (J.-H.J.)

**Keywords:** heat stress, THI, cool water, laying hens, laying performance

## Abstract

We used an animal welfare-certified open-type layer farm and analyzed the egg production, egg quality, rectal temperature, and yolk corticosterone levels of laying hens supplied with cool water during the summer season (avg. 33 ± 3.89 °C). A total of 5750 Hy-Line Brown laying hens at 53 weeks of age were used, and two treatment groups were established: a control group (2900 hens) and a cool water treatment group (2850 hens). The water temperature of the control group was 25.3 ± 0.8 °C and the cool water was 20.1 ± 0.3 °C; all other environment parameters (lighting, ventilation, temperature, feed, etc.) were the same. The experiment was conducted for a total of 9 weeks (between July and September 2024), and during this period, the temperature–humidity index (THI) inside the breeding facility averaged 85.21, which corresponds to the cool water supply range (80 < THI < 90). As a result, the cool water treatment group maintained high productivity and showed low mortality (*p* < 0.05). In addition, hens provided with cool water showed high eggshell strength and low yolk corticosterone levels (*p* < 0.05). The core finding of this study is that the supply of cool water in summer is effective in maintaining the productivity and egg quality of laying hens and reducing HS. This is significant in that it suggests it is possible to manage laying hens in summer in a simple way, and it can also be used as basic data for designing future studies, such as using a combination of natural products including vitamins and minerals.

## Simple Summary

In order to maintain and improve the economic efficiency of the poultry industry, it is essential to find countermeasures for the high-temperature environment in summer. Excessive heat exposure reduces feed intake of poultry and reduces the quality of livestock products; in particular, the characteristics that make it difficult to dissipate heat cause high stress. Providing cool water helps regulate body temperature and is considered to be beneficial for reducing stress and maintaining productivity, but there have been no studies that actually verified the effectiveness of this practice on a farm. Therefore, we analyzed the differences in egg production, egg quality, and stress levels when cool water was supplied in a high-temperature environment on laying hen farms. Providing cool water helped maintain the productivity and egg quality of laying hens even under high-temperature conditions. In addition, providing cool water is an effective way to alleviate heat stress (HS).

## 1. Introduction

The poultry industry provides an excellent source of protein worldwide, including meat and eggs, but is currently facing serious challenges due to heat stress (HS) caused by high temperatures during the summer [1]. According to a report, the US suffers losses of USD 128–165 million/year due to HS [2]. The impact of HS is expected to increase further, especially as the intensity, duration, and frequency of heat waves continue to increase due to global warming [2].

HS negatively impacts chickens at all life stages, from young chicks to adult birds [3]. Research has demonstrated that exposure to excessive heat leads to adverse effects, including a reduction in feed intake, lower egg production, diminished weight gain, poor egg quality, and increased mortality rates [4,5,6,7]. Elevated temperatures are known to activate the hypothalamic appetite center, triggering neural signals that suppress feeding behavior [8]. It has been reported that for every 1 °C rise above the thermoneutral range (20 to 25 °C), feed consumption decreases by approximately 2% in poultry [9,10]. In addition, oxidative stress caused by HS can cause an imbalance of pro-oxidants and antioxidant systems in the body and affect egg quality through mitochondrial dysfunction and an increased concentration of lipid and protein oxidation products, for example [11]. Cruvinel et al. [12] reported that HS causes electrolyte imbalances, which, in turn, impair the absorption of minerals important for maintaining egg and bone quality due to altered acid–base homeostasis. Unlike many animals that regulate body temperature through sweat gland activity and evaporative cooling via the skin, chickens lack sweat glands due to their feather coverage, making heat dissipation more challenging [13]. Consequently, they primarily rely on evaporative cooling through respiration to regulate body heat. Although this mechanism is effective, it becomes a disadvantage during extreme heat conditions. The optimal thermoneutral zone for chickens falls between 19 and 22 °C, allowing for sufficient sensible heat loss to maintain their normal body temperature, which ranges from 40.6 to 42.4 °C [14].

The temperature–humidity index (THI) is a widely used environmental indicator for predicting production losses in livestock exposed to hot and humid conditions [15]. The THI has been developed to assess the impact of the thermal environment on the thermoregulatory status of animals and is used to prevent the negative aspects of HS [15]. The THI comprises a linear combination of dry-bulb (T_db_) and wet-bulb (T_wb_) temperatures [16], and the THI classifies data into normal, alert, danger, and emergency zones. When chickens experience temperatures beyond their thermal comfort range, they exhibit various signs of HS, including panting, prostration, wing spreading, and increased water intake [17]. Panting, which involves rapid, open-mouth breathing, facilitates evaporative cooling through the respiratory system. However, if this mechanism alone is insufficient to dissipate excess heat, chickens may suffer from severe HS, which can ultimately lead to mortality [18].

However, if cooler water intake encourages increased hydration, it can enhance respiratory evaporative cooling, resulting in a meaningful thermoregulatory benefit [19]. Additionally, we propose that offering cool water may positively influence feed intake, thereby improving eggshell quality. Water is a nutrient with several positive benefits, and is the most crucial and essential nutrient for overall health. Water aids in the regulation of body temperature, feed digestion and absorption, nutrient transfer, and waste disposal from the body [3]. Usually, under average normal ambient temperature, the birds consume water approximately 1.6 to 2.0 times more than feed based on their weight [20]. During HS, water consumption quadruples to increase heat dissipation via convection, conduction, and radiation [19]. Abioja et al. [21] reported that it would be beneficial if the temperature of the water consumed was lower than the body temperature. Beker and Teeter [20] also showed improved feed consumption and body weight in broilers as drinking water temperature decreased by 10 °C from 43.3 °C in a high-temperature environment. For these reasons, poultry farmers have attempted to use ice cubes or provide cool water in hot environments [21], but there is limited research to support this, and, in particular, no studies have investigated the practical effectiveness of these practices on farms.

Therefore, in this study, we aimed to analyze the differences in egg production, egg quality, stress concentrations, and rectal temperature by providing drinking water of different temperatures to laying hens in a high-temperature environment. The water temperatures compared were 25 °C [22], which was the average temperature in the chicken house, and 20 °C, which is 5 °C lower. The originality of our study is that it is the first of its kind to be conducted as a field trial in a summer laying hen farm. The presentation of these results may help farm managers to alleviate HS and maintain productivity in hot environments.

## 2. Materials and Methods

The protocol for this experiment was reviewed and approved by the Institutional Animal Care and Welfare Committee of the National Institute of Animal Science, Rural Development Administration, Republic of Korea (NIAS 2022-0565).

### 2.1. Experimental Design

This study was conducted in an animal welfare-certified free-range housing system of laying hens located in Okcheon-gun, Chungcheongbuk-do, using 5750 Hy-Line Brown laying hens at 53 weeks of age. The information on the experimental farm in this study, such as the area of the house size, stocking density, and number of feeders, waterers, and perches, is summarized in Table 1.

**Figure 1:**
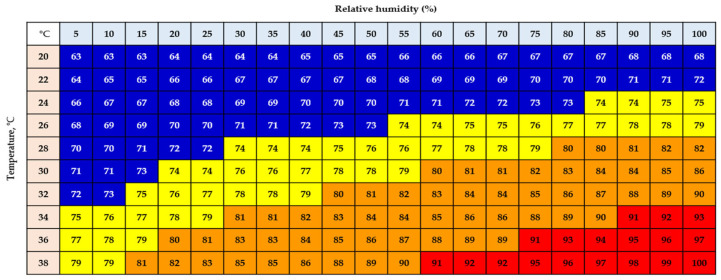
Temperature–humidity index for laying hens. Bird normal zone = THI < 73 (blue color); alert zone = THI 74 to 79 (yellow color); danger zone = THI 80 to 90 (orange color); emergency zone = THI > 90 (red color). Cool water supply THI range: 80 < THI < 90.

In this study, two buildings of the same size were used: one building was designed as a control group (2850 hens), and the other building was designed as a treatment group (2900 hens). Each building was divided into five equal sections (replicates) from the front to the back, and an equal number of eggs and laying hens were selected from each section for analysis. The experimental period was selected during the summer season, which has the hottest weather; this was determined by reviewing the most recent summer weather information reported [23,24,25]. As a result, the experiment period was conducted for a total of 9 weeks, from 9 July to 3 September 2024. The temperature inside and outside the house was measured using a temperature and humidity data logger (testo 176 H1, Testo SE & Co. KGaA, Titisee-Neustadt, Germany). The average ambient temperature investigated in our study was 33 ± 3.89 °C, with a maximum of 39.7 °C and a minimum of 22.4 °C. And also, the average internal temperature of the poultry house was 32.8 ± 3.23 °C, with a maximum of 41.9 °C and a minimum of 23.2 °C. In this study, each drinking water source was supplied when the THI was 80 to 90 (Figure 1), and the THI of the chicken house during the experimental period was 85.21, which was within the range of cool water supply (Table 2). The THI level at this time was checked in real time through the control panel, and the cool water supply was designed to be stopped if it exceeded the THI level we set.

**Figure 2:**
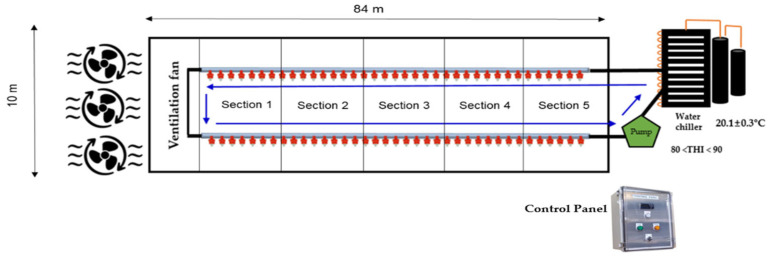
Internal schematic diagram of the breeding facility (cool water treatment) of a laying hen experimental farm. Schematic representation of the sampling locations (Sections 1~5) where egg production and rectal temperature were measured.

An internal schematic diagram of the selected experimental farm is shown in Figure 2. The water chiller operates according to the set THI range (80 < THI < 90) to ensure that the laying hens in the cool water treatment area were consuming cool water at an appropriate temperature. The temperature of the supplied cool water was 20.1 ± 0.3 °C, while the drinking water temperature in the control group was 25.3 ± 0.8 °C.

A commercial-type basal diet was formulated to meet or exceed the Korean feeding standard for layers [26]. Table 3 shows the composition and calculated chemical composition values of basic feeds based on corn and soybean meal. During the experimental period, all laying hens were provided with feed and water ad libitum and were exposed to a 16 h/8 h light/dark cycle.

### 2.2. Laying Performance and Economic Evaluation

In terms of laying performance, the analysis items were egg production, egg weight, feed intake, feed conversion ratio (FCR), and mortality rate. The egg collection time was 10:00 a.m. every day, which was the time when the farmer consistently collected their eggs. Egg production rate was recorded in hen–day format (considering the number of laying hens and the number of eggs on that day). Egg weight and mortality were also observed daily. Feed intake was measured by checking the measuring device in the feed bin, and the provided and remaining feed were recorded at one-week intervals. Based on this, the feed intake for one week by treatment group was calculated, and this was converted and expressed as daily feed intake, which is the amount of feed eaten by one bird per treatment group per day. The feed conversion ratio (FCR) was calculated by the following formula, referring to the study of Kim et al. [27]:

$$\mathrm{FCR}=\frac{g\;\mathrm{of}\;\mathrm{feed}\;\mathrm{consumed}}{g\;\mathrm{of}\;\mathrm{egg}\;\mathrm{mass}}$$

The economic evaluation was converted and compared based on the cool water treatment group (2900 birds) and was calculated with reference to the analysis method of Han et al. [28]. In summary, the economic feasibility analysis was conducted based on the sales amount generated from the eggs, and the calculation was made by considering the laying rate and egg weight of each treatment group. Accordingly, the average egg weight of both treatment groups was in the extra-large egg range (egg weight: 60–66 g), and the price of one extra-large egg in Korea in 2025 was KRW 178 (South Korean currency, won). The economic benefits were calculated in KRW and United States dollars (USD) by comparing the monthly egg sales of each treatment, with the exchange rate set at USD 1.00 = KRW 1200. The installation cost of the machine used in this study was KRW 500,000, and depreciation was expected to take 10 years.

### 2.3. Determination of Egg Quality Parameters

Egg quality analysis was conducted 4 times (at 0, 2, 5, and 7 weeks) during the experimental period. A total of 60 eggs (30 eggs per group (6 eggs per section)) were randomly collected and used each time. Each egg was weighted and then broken open for quality analysis. Eggshell strength was measured by a compression test cell with Texture systems (Model T2100C, Food Technology Co., Ltd., Rockville, MD, USA) and was expressed as the unit of compression force exposed to the unit eggshell surface area (in kilograms per square centimeter). Eggshell thickness was defined as the mean value of measurements at 3 different locations on the egg (air cell, equator, and sharp end) and was measured with a dial pipe gauge (model 7360, Mitutoyo Co. Ltd., Kawasaki, Japan) and calculated using the following formula by Kang et al. [29]:

Eggshell thickness = (sharp point thickness + equator thickness + air cell thickness)/3(1)

Egg yolk color was evaluated by the Roche color fan (Hoffman-La Roche Ltd., Basel, Switzerland; 15 = dark orange; 1 = light pale). Eggshell color was evaluated by the eggshell color fan (Samyang Co. Ltd., Seoul, Korea). Egg albumen height (mm), one of the indicators for determining Haugh unit level, was measured using a micrometer (model S-8400, Ames, Walthman, MA, USA) immediately after breaking onto a plate on a flat surface [30]. Then, the Haugh unit value was calculated using the following formula described by Eisen et al. [31] based on the egg weight and albumen height.

Haugh unit = 100 log(H – 1.7W^0.37^ + 7.6) (2)

where H is the egg albumen height (mm) and W is the egg weight (g).

### 2.4. Measurement of Rectal Temperature

On weeks 0, 2, 5, and 7, rectal temperatures were measured in 10 randomly selected hens per group (2 laying hens selected per section). For the rectal temperature analysis, a digital thermometer (Eurogiant emergency digital thermometer, model 2022, EuroGeneral, Dublin, Ireland) was used, and the probe of the thermometer was inserted approximately 3 cm deep into the rectum of selected laying hens and held for approximately 30 s until a steady reading was obtained. Temperature measurements were measured and recorded to 0.1 °C [32].

### 2.5. Corticosterone in Yolk Samples

Yolk corticosterone concentrations were measured at 0, 2, 5, and 7 weeks, and 4 g of pooled yolk was vortexed with an equal volume of PBS. Then, 1 mL of the yolk suspension was mixed with an equal volume of ethanol, incubated at 37 °C for 1 h, and subsequently centrifuged [33]. Then, 50 µL of the supernatant was mixed with 50 µL of ethanol and 50 µL of PBS solution, and these mixtures were analyzed with a corticosterone ELISA kit (Enzo life science Inc., ADI-901-907, Farmingdale, NY, USA), as previously described [34,35]. At this time, 10 replicates were performed per treatment for each week.

### 2.6. Statistical Analysis

In this study, we attempted to remove outliers and missing values by checking the homogeneity of variance and normality of data. If the residuals were more than three times the standard error of the parameter, they were considered outliers to be excluded from the data set before statistical analysis. Subsequently, all data except mortality data, which were not continuous variables, were analyzed using analysis of variance (ANOVA) in a completely randomized design in statistical analysis system (SAS) software (version 9.4; Statistical Analysis System Institute Inc., Cary, NC, USA) through the Proc Mixed procedure. Mortality data were analyzed using the chi-squared test. Outliers were checked with the UNIVARIATE procedure in SAS; however, none were identified. Differences among least-squares means were assessed using the PDIFF option with *t*-test.

Here, each section of the rearing facility was an experimental unit for the analysis of egg production parameters. Data were collected for all experimental periods and the results are described in the following sections. Egg quality items and yolk corticosterone levels were considered as the experimental units for the number of eggs analyzed and were statistically analyzed from the start to the end date. For rectal temperature measurements, individual chickens selected from each section were used as experimental units. A macro program was utilized to categorize output values into letter groups. Statistical significance was defined at *p* < 0.05, with trends considered in the range 0.05 ≤ *p* ≤ 0.10.

## 3. Results

### 3.1. Laying Performance

Table 4 shows the results of egg productivity differences when providing drinking water of different temperatures to laying hens in a high-temperature environment. The hen day egg production (*p* < 0.05) over the entire experimental period was higher in the cool water treatment than in the control, and there was also a significant difference in feed intake (*p* < 0.05). There was no difference in egg weight and FCR between treatment groups (*p* > 0.05). Mortality over the whole experiment period was lower in the cool water treatment than in the control (*p* < 0.05). In the economic comparison results for the same number of individuals (2900 birds), the number of eggs produced per month was 870 more in the cool water treatment group, and the economic benefit was KRW 154,860 (USD 129.05) higher.

### 3.2. Egg Quality

Table 5 shows the results of comparing egg characteristics when drinking water of different temperatures was provided to laying hens in a high-temperature environment. The Haugh units showed no significant difference despite the provision of cool water (*p* > 0.05). However, five weeks after the start of the experiment, the treatment group that drank cool water produced harder eggs than the control group (*p* < 0.05). Cool water supply did not affect other analysis items such as eggshell thickness and egg yolk color (*p* > 0.05).

### 3.3. Rectal Temperature

**Figure 3:**
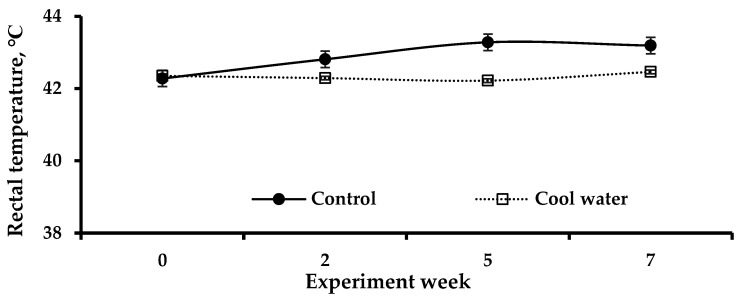
Changes in rectal temperature due to cool water supply under high-temperature environmental conditions. Control drinking water temperature, 25.3 ± 0.8 °C; cool drinking water temperature, 20.1 ± 0.3 °C.

Figure 3 summarizes the changes in rectal temperature that occur when water of different temperatures is provided in a high-temperature environment. The supply of cool water (20.1 ± 0.3 °C) did not have any significant effect on reducing rectal temperature in laying hens in a high-temperature environment (*p* > 0.05).

### 3.4. Corticosterone in Egg Yolk

**Figure 4:**
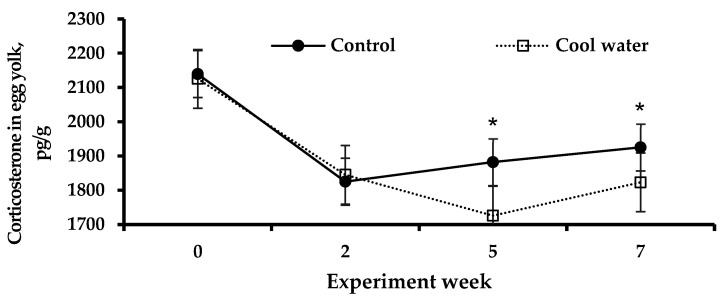
Changes in corticosterone concentrations in egg yolk due to cool water supply under high-temperature environmental conditions. Control drinking water temperature, 25.3 ± 0.8 °C; cool drinking water temperature, 20.1 ± 0.3 °C. Significant: (*) = *p* < 0.05.

Changes in egg yolk corticosterone levels in a high-temperature environment due to the provision of cool water are shown in Figure 4. There was no significant difference in the corticosterone levels in the egg yolk between the treatment groups at 0 and 2 weeks (*p* > 0.05). However, it was confirmed that the provision of cool water significantly reduced the corticosterone levels compared to the control at 5 and 7 weeks (*p* < 0.05).

## 4. Discussion

The notion that a high-temperature environment causes stress in laying hens and reduces productivity has already been reported in many studies [3,35,36,37,38]. It has been shown that laying hens in high-temperature environments had a 30% decrease in feed intake and an 11% decrease in egg production [27]. Poultry effectively regulate their body temperature in high-temperature environments by panting (evaporative cooling), and in this process, water intake increases and feed intake decreases [3]. Moreover, laying hens in high-temperature environments suffer from heat-induced intestinal damage, inhibiting nutrient intake, digestibility, and intestinal absorption and limiting the availability of nutrients essential for egg formation [35]. Mashaly et al. [39] reported that broilers and laying hens raised in high-temperature environments (35 °C) had higher mortality rates than poultry raised in thermoneutral conditions, and for this reason, high-temperature environments reduce productivity. On the one hand, providing cool water can lower body temperature through conduction and convection, which may prevent decreased feed intake [3]. Preventing a decrease in feed intake helps to ensure sufficient calcium intake, which is necessary for egg formation and, in turn, increases egg production [3,40]. Silva et al. [41] reported that livestock farms with good thermal isolation from the external environment, such as tunnel ventilation and cooling pads, improved productivity (12.04%) and reduced mortality (30–40%), which contributed to the economic performance of farms. In our study, the treatment group provided with cool water also showed higher feed intake and egg production and lower mortality than the control group. This suggests that the supply of cool water helps maintain the body temperature of laying hens in hot environments, allows them to consume feed normally, and creates a more comfortable housing environment.

In high-temperature environments, the supply of cool water has been shown to maintain eggshell strength. In a study by Glatz [42], eggshells were thicker and heavier regardless of the breed of laying hens when cool drinking water was provided in a hot environment. In a similar study, it was found that laying hens exposed to HS had progressively thinner eggshells and reduced eggshell weight over time [36]. Mahoud et al. [43] confirmed that a high-temperature environment affects the Ca^2+^ circulation pattern in laying hens and reduces the transport of calcium and absorption capacity [43]. On the other hand, the characteristic poor eggshell quality of laying hens affected by HS may be due to reduced feed intake [44]. The production of good-quality eggshells by laying hens depends on the availability of Ca in the feed and skeletal reserves [44]. Reduced feed intake limits the availability of Ca in the blood for eggshell formation [45], and low levels of Ca intake can lead to bone resorption and hyperphosphatemia [44]. Chauhan and Roy [46] reported that hyperphosphatemia causes more blood to be distributed to the skin to regulate body temperature, which results in decreased Ca supply to the uterus and suppression of CaCO_3_ formation in the uterine glands. These mechanisms can sufficiently support the results showing higher feed intake and eggshell thickness in the treatment group provided with our cool water. Providing cool water allows laying hens to consume sufficient feed and utilize nutrients normally in a high-temperature environment, thereby producing good-quality eggs. On the other hand, it has been reported that HS in a high-temperature environment can affect not only the indicators analyzed in our study but also the specific gravity, albumen thickness, and yolk pH of eggs [1,47,48]. From this perspective, further studies and analyses targeting several indicators are necessary to clearly verify the effect of providing cool water on improving egg quality.

Measuring rectal temperature is a method used to assess the response of poultry to environmental stress and serves as an indicator of thermal homeostasis [32]. HS affects respiratory alkalosis, increasing body temperature and panting rate and impairing growth performance [32]. Therefore, if poultry are panting (with an increased respiratory rate) and have a high rectal temperature, it indicates that they are experiencing unfavorable environmental stress, such as that induced by a high-temperature environment [49]. Meanwhile, providing cooling devices has been shown to reduce rectal temperature in poultry [35,50]. Hu et al. [51] found that providing cooled perches allowed conductive heat loss from the feet of laying hens to cool water, improving their thermoregulatory capacity and increasing thermal comfort and welfare. In a study by Abioja et al. [52], cool water (8 °C) supply resulted in lower rectal temperatures compared to water at a normal temperature (29.5 °C). However, under HS conditions, cool water (16.4 °C) did not reduce the rectal temperature of broilers [53]. Meanwhile, Abioja et al. [52] reported that the level of water’s ability to act as a heat sink varies depending on the water temperature, degree of heat exposure, and breed of poultry. From this perspective, there was no difference in rectal temperature even though cool water at a temperature of about 20.1 °C was supplied, which seems to be insufficient for ensuring the thermal comfort of laying hens. Therefore, future research is needed to explore the water temperature that can effectively alleviate thermal stress in laying hens.

Measuring corticosterone levels in various samples, including blood, feces, feathers, and egg yolk, is a widely used approach for assessing biological stress indicators in birds [34,54,55,56]. Analysis using these methods is a key technique for measuring corticosterone, a stress biomarker in poultry [57,58,59]. However, given the challenges of sample collection and analysis (feces), the acute stress caused (blood), and the invasive methods required (blood, feathers), it may be more effective to use eggs to assess stress levels rather than feces, blood, and feathers [54,57,58,59,60]. In particular, collecting blood to conduct a stress level analysis is a method used in many studies, but it should be undertaken with caution, as it depends on the level of animal handling by the sample collector [61]. Meanwhile, previous studies [58,59] have utilized corticosterone levels in the albumen and yolk of chicken eggs as a noninvasive method for assessing stress. During the egg formation process, substances and hormone concentrations present in the bloodstream are incorporated into the yolk. An increase in blood corticosterone levels leads to its subsequent transfer into the egg [58]. Therefore, in this study, corticosterone concentrations were analyzed using egg yolks as a noninvasive alternative. Our results showed that egg yolk corticosterone concentration was significantly increased in the control group at the time of the experiment and at 5 weeks of age. In support of this, Farghly et al. [62] suggested that providing cool water in a high-temperature environment was effective in reducing corticosterone levels in turkeys. In addition, in the study by Park et al. [63], providing water at 9 °C was more effective at reducing stress in broilers in an extreme HS environment than providing water at 14 °C. However, our study has limitations in that it investigated corticosterone levels using only egg yolk and there are very few cases in which changes in stress hormones were studied according to the provision of cool water. Therefore, research is required to compare changes in stress hormones using various samples (blood, feces, feathers, etc.) and to confirm the optimal water supply temperature for stress reduction.

## 5. Conclusions

The productivity indicators of laying hens were significantly affected by cool water supply in conditions of HS. Providing cool water for 9 weeks resulted in increased egg production, increased feed intake, and significantly improved eggshell strength, resulting in improved overall egg production and quality. Variations in egg quality, like eggshell strength, could be attributed to increased feed consumption. Rectal temperature did not show significant differences among treatments, but the concentration of corticosterone in egg yolk was significantly reduced in the cool water group compared with the control. In conclusion, the provision of cool water under HS conditions in summer has a positive effect on the productivity of laying hens and may improve the welfare and productivity of hens in the face of climate change.

## Figures and Tables

**Table 1:** Summary information about the experimental farm (per building).

Items		Information
Farm		Animal welfare-certified farm
Region		Okcheon-gun, Chungcheongbuk-do, Republic of Korea
Strain		Hy-Line Brown
Housing type		Free-range system
Construction materials		Sandwich panel
Ventilation system (type)		Mechanical ventilation system (tunnel-type)
Flock size		2800~2900 hens
House size, m, m^2^		10 × 84, 840
Stocking density, hens/m^2^		7.5
Number installed	Feeders	239
	Waterers	805
	Perches (length, m)	42 (19.2), 2 (28.4)

**Table 2:** Information on temperature conditions in laying hen housing during the study period.

Items	Levels
Outside average temperature, °C	33.5 ± 3.89
Max temperature, °C	39.7
Min temperature, °C	22.4
Inside average temperature, °C	32.83 ± 3.23
Max temperature, °C	41.9
Min temperature, °C	23.2
Average THI	85.21

**Table 3:** Composition and nutrient content of experimental diets.

Items	Composition
Ingredients, g/kg	
Corn	411.5
Wheat	150.0
Soybean meal	250.0
Corn distiller’s dried grains with solubles	50.0
Canola meal	20.0
Tallow	5.0
Molasses	5.0
Limestone	97.0
Dicalcium phosphate	7.0
Sodium chloride	2.0
Vitamin premix ^1^	1.5
Mineral premix ^2^	1.0
Total	1000.0
Calculated nutrient composition ^3^	
ME, MJ/kg	11.32
Crude protein, g/kg	186.0
Available phosphate, g/kg	3.3
Lysine, g/kg	9.7
Methionine, g/kg	3.1

^1^ Provided per kilogram of the complete diet: vitamin A (vitamin A acetate), 12,500 IU; vitamin D_3_, 2500 IU; vitamin E (DL-α-tocopheryl acetate), 20 IU; vitamin K_3_, 2 mg; vitamin B1, 2 mg; vitamin B_1_, 2 mg; vitamin B_2_, 5 mg; vitamin B_6_, 3 mg; vitamin B_12_, 18 μg; calcium pantotenate, 8 mg; folic acid, 1 mg; biotin 50 μg; niacin, 24 mg. ^2^ Provided per kilogram of the complete diet: Fe (FeSO_4_·7H_2_O), 40 mg; Cu (CuSO_4_·H_2_O), 8 mg; Zn (ZnSO_4_·H_2_O), 60 mg; Mn (MnSO_4_·H_2_O), 90 mg; Mg (MgO) as 1500 mg. ^3^ Nutrient contents in all diets were calculated.

**Table 4:** Comparison of production and cost benefit of laying hens in a high-temperature environment according to drinking water temperature. ^1^

Items	Treatments ^2^		*p*-Value
	Control	Cool Water	
Hen day egg production, %	83.5 ± 1.8 ^b^	84.5 ± 0.9 ^a^	0.04
Feed intake, g/birds	125.9 ± 2.5 ^b^	129.8 ± 2.3 ^a^	0.03
Egg weight, g	63.1 ± 0.39	63.9 ± 0.47	0.39
FCR, g/g	2.39 ± 0.01	2.40 ± 0.01	0.28
Total mortality, %	2.39 ^a^	1.55 ^b^	<0.01
Economic gain per 2900 laying hens/month			
Number of eggs produced, n	72,645	73,515	-
Egg sales revenue, KRW	12,930,810	13,085,670	-
Egg sales revenue, USD	10,775.68	10,904.73	-

^a^, ^b^ Means in the same row with different superscripts differ significantly (*p* < 0.05). ^1^ Data are least-squares means of 5 replicates per treatment. ^2^ Standard error of means.

**Table 5:** Comparison of egg quality changes according to drinking water temperature in high-temperature environment laying hens. ^1^

Items	Treatments ^2^		*p*-Value
	Control	Cool Water	
Haugh unit			
0 weeks	85.67 ± 8.02	85.17 ± 5.73	0.781
2 weeks	86.75 ± 6.64	88.30 ± 3.71	0.267
5 weeks	86.13 ± 7.12	88.70 ± 5.99	0.136
7 weeks	86.78 ± 6.68	88.70 ± 5.32	0.226
Eggshell strength, kgf			
0 weeks	4.43 ± 0.66	4.15 ± 1.01	0.210
2 weeks	3.87 ± 1.22	3.93 ± 1.28	0.839
5 weeks	4.13 ± 1.02 ^b^	4.59 ± 0.64 ^a^	0.042
7 weeks	4.07 ± 0.93 ^b^	4.45 ± 0.55 ^a^	0.047
Eggshell thickness, mm			
0 weeks	0.374 ± 0.026	0.378 ± 0.029	0.550
2 weeks	0.394 ± 0.026	0.397 ± 0.030	0.677
5 weeks	0.395 ± 0.029	0.390 ± 0.028	0.506
7 weeks	0.382 ± 0.030	0.383 ± 0.020	0.801
Eggshell color			
0 weeks	27.32 ± 3.46	28.79 ± 3.69	0.115
2 weeks	28.32 ± 3.11	27.96 ± 3.06	0.653
5 weeks	28.50 ± 4.26	27.75 ± 4.33	0.506
7 weeks	30.30 ± 4.57	27.86 ± 3.20	0.320
Egg yolk color			
0 weeks	6.85 ± 0.67	6.72 ± 0.46	0.360
2 weeks	6.27 ± 0.42	6.62 ± 0.46	0.103
5 weeks	6.61 ± 0.51	6.46 ± 0.47	0.222
7 weeks	6.81 ± 0.42	6.61 ± 0.42	0.073

^a^, ^b^ Means in the same row with different superscripts differ significantly (*p* < 0.05). ^1^ Data are least-squares means of 30 eggs per treatment. ^2^ Standard error of means.

## Data Availability

The original contributions presented in this study are included in the article. Further inquiries can be directed to the corresponding author.
